# Stop feeling: inhibition of emotional interference following stop-signal trials

**DOI:** 10.3389/fnhum.2013.00078

**Published:** 2013-03-14

**Authors:** Eyal Kalanthroff, Noga Cohen, Avishai Henik

**Affiliations:** Department of Psychology, Zlotowski Center for Neuroscience, Ben-Gurion University of the NegevBeer Sheva, Israel

**Keywords:** emotional processing, executive functions, inhibitory control, stop signal, SSRT, emotional interference

## Abstract

Although a great deal of literature has been dedicated to the mutual links between emotion and the selective attention component of executive control, there is very little data regarding the links between emotion and the inhibitory component of executive control. In the current study we employed an emotional stop-signal task in order to examine whether emotion modulates and is modulated by inhibitory control. Results replicated previous findings showing reduced inhibitory control [longer stop-signal reaction time (SSRT)] following negative, compared to neutral pictures. Most importantly, results show decreased emotional interference following stop-signal trials. These results show that the inhibitory control component of executive control can serve to decrease emotional effects. We suggest that inhibitory control and emotion have a two-way connection in which emotion disrupts inhibitory control and activation of inhibitory control disrupts emotion.

## Introduction

Emotional stimuli play a major role in human lives. They are considered to receive prioritized processing and therefore affect behavior, cognition, and physiology. Maladaptive emotional processing and deficient emotion regulation are core factors in different psychopathologies and therefore it is highly important to understand their nature. One of the most studied topics among emotion scientists is the relationship between emotion and attention (e.g., Vuilleumier, 2005; Pessoa, 2009). Emotional stimuli are considered to capture attention and hence disrupt performance (i.e., elongate RT) in various tasks, such as simple discrimination tasks (Hartikainen et al., 2000; Buodo et al., 2002). This effect is termed emotional interference [i.e., elongated reaction time (RT) for negative compared to neutral trials]. Recently, a vast amount of cognitive and affective studies has been dedicated to exploring the influence of emotional information on tasks that require executive control. Executive control is considered to be a “high” order system that incorporates several attentional subsystems (Posner and Petersen, 1990; Verbruggen and Logan, 2008; Banich, 2009), which act together in order to guide behavior in accordance with internal goals (Shallice and Norman, 1986; Miyake et al., 2000; Miller and Cohen, 2001; Banich, 2009). Importantly, both emotion and executive control are crucial elements in goal-directed behavior. Therefore, studying the links between these two systems is important for understanding adaptive and maladaptive behavior. The aim of the current study is to investigate the connection between inhibitory control—a component of executive control—and emotion eliciting stimuli^1^ (i.e., negative pictures).

The influence of emotional stimuli on executive control was studied mainly using selective attention tasks such as the Stroop (1935) and the flanker (Eriksen and Eriksen, 1974) tasks. These tasks measure the ability to attend to a relevant dimension and ignore irrelevant, distracting information. The findings obtained from studies that used such tasks are inconsistent. For instance, using a modified version of the flanker task, Dennis et al. (2008) found reduced executive control following presentation of fearful faces. Similarly, Padmala et al. (2011) found that negative stimuli reduced conflict monitoring and concluded that there are shared resources between executive control and emotional processing. Other studies found the opposite effect; namely, compared to neutral information, emotional information improved executive control in selective attention tasks (e.g., Kanske and Kotz, 2010, 2011a,b; for further discussion see Cohen and Henik, 2012; Kanske, 2012).

Although the effects of emotion on executive control have been widely studied, only recently have researchers begun to explore the effects of executive control on emotion. Okon-Singer et al. (2012) suggested that attentional factors, such as executive control, can exert top-down modulation on emotion (see also Bishop, 2008, for the neural mechanism responsible for regulating attention to threat-related cues; Ochsner and Gross, 2005, for a review). This top-down modulation is crucial in situations in which the emotional information is irrelevant and can disrupt goal-directed behavior. In line with this suggestion, neuroimaging studies found that activation of brain regions involved in executive control (prefrontal, especially dorsolateral, and parietal cortex) attenuates the activation in brain regions involved in emotional processing (mainly the amygdala) (Hariri et al., 2000; Liberzon et al., 2000; Pessoa, 2005; Vuilleumier, 2005; Etkin et al., 2006; Blair et al., 2007; Mitchell et al., 2008; Hart et al., 2010). There is also behavioral evidence for the top–down regulation of emotional stimuli. Etkin et al. (2006, 2010) used an emotional Stroop-like task and found a conflict adaptation effect (i.e., emotional conflict in the current trail was attenuated following a conflict in the previous trail). The authors concluded that executive processes (i.e., selective attention) can attenuate emotional response. Recently, we found more direct behavioral evidence for the top–down regulation of emotional stimuli (Cohen et al., 2011, 2012). We presented negative and neutral pictures following a flanker target and measured emotional interference in a following discrimination task. Participants were required to respond to the direction of a middle arrow and ignore flanking arrows. The flanking arrows could be congruent (<<<<<) or incongruent (<<><<) with the target arrow. Incongruent trials consist of a conflict and are considered to recruit executive control processes (as indicated in elongated RT in incongruent vs. congruent trials). Emotional interference was present after congruent trials, but was eliminated after incongruent trials (Cohen et al., 2012; see also Blair et al., 2007).

Looking at selective attention tasks to examine the connection between emotion and executive control might be insufficient. In line with this notion, many researchers suggested that executive control is not unitary and urged discerning between different components of control (Rafal and Henik, 1994; Harnishfeger, 1995; Miyake et al., 2000; Nigg, 2000; Banich, 2009). Selective attention tasks, such as the Stroop (Stroop, 1935; MacLeod, 1991) and the flanker (Posner and Petersen, 1990) tasks measure the ability to attend to a relevant dimension and ignore irrelevant, distracting information. The ability to ignore irrelevant information might involve inhibition (Verbruggen et al., 2004; Kalanthroff et al., 2012)—a core component of executive control (van Veen and Carter, 2006; Verbruggen and Logan, 2008). In the current paper we ask whether the inhibitory component of executive control is influenced and can modulate emotional reaction in a similar way as is found in selective attention.

### Inhibitory control

An important ingredient of executive control, and perhaps a hallmark of it, is the suppression of irrelevant information, thought, or action (van Veen and Carter, 2006; Verbruggen and Logan, 2008). This component of executive control is termed inhibitory control and is commonly associated with activation in the right inferior frontal gyrus (rIFG; Aron et al., 2003). To study this process in the laboratory, consider the stop-signal task (Logan and Cowan, 1984; Logan, 1994), which examines the ability to suppress an already initiated action that is no longer appropriate. In the classic task, participants are asked to address a visual stimulus (go signal) with a motor response as fast as possible. In about one fourth of the trials, an auditory stimulus (stop signal), which signals to participants to inhibit their motor response, comes right after the visual go signal. The duration between the go signal and the stop signal (stop-signal delay; SSD) is submitted to a tracking procedure and changes from one trial to the next based on the participant's success in inhibiting his or her response (i.e., a successful inhibition will cause the next trial to be more difficult—the SSD will be longer—and vice versa). Eventually, it is possible to estimate the stop-signal reaction time (SSRT), which is the time needed for successful inhibition. SSRT has proven to be an important measure of cognitive control (Verbruggen and Logan, 2008). Logan and Cowan (1984) and Logan et al. (1984) compared the performance in the stop-signal task to a horse race between the more automatic go process, triggered by the presentation of the go signal, and the executive stop process, triggered by the stop signal. Logan et al. (1984) argued that “response inhibition phenomena are consistent with a hierarchical theory of attention in which a high level process determines the significance of incoming stimuli and decides whether to abort the current stream of thought and action or to queue the new stimuli along with the old ones, to be processed as resources become available” (p. 290).

### Inhibitory control and emotion

Few studies concentrated on the relationship between emotion and inhibitory control. Using a modified version of the stop-signal paradigm, Verbruggen and De Houwer (2007) found that emotional stimuli (negative or positive pictures) decrease the efficiency of inhibitory control (i.e., longer SSRT in emotional trials). Sagaspe et al. (2011) found prolonged RT in the presence of incidental threatening information, though SSRT was unaffected by emotion. However, these researchers did find that neural circuits engaged by inhibition are modulated by threatening information. Specifically, they found that stopping in a threatening trial was associated with activations in the orbitofrontal cortex (and not the inferior frontal gyrus usually associated with stopping). This finding implies that inhibitory control in the presence of emotional information may be different from inhibitory control in neutral situations. Pessoa et al. (2012) used the stop-signal task with high- and low-threat stimuli and found that the efficiency of inhibitory control is increased by low-threat stimuli and decreased by high-threat stimuli. This finding is in line with Pessoa et al. (2012) notion that low threat improves executive control since it increases goal-directed behavior (see also Kanske, 2012), whereas high threat attracts resources available for the task and hence disrupts executive processes.

In contrast to the mixed findings regarding the influence of emotion on selective attention, with respect to inhibitory control most findings are consistent regarding the disruptive influence of emotion on inhibitory control. A previous study showed that an emotional stimulus deteriorates performance of both go and stop processes (Verbruggen and De Houwer, 2007). However, it is not yet clear whether inhibitory control exerts a regulatory effect on emotion, similar to the effect of selective attention. This question is highly important for few reasons: (a) understanding the connection between inhibition and emotion influence on performance can deepen our knowledge regarding the connection between “high” (e.g., executive control) and “low” (e.g., emotion) cognitive systems. (b) Deficient inhibitory control underlies different psychopathologies and mood disorders, such as autism (e.g., Geurts et al., 2004), schizophrenia (e.g., Enticott et al., 2008), obsessive-compulsive disorder (e.g., Chamberlain et al., 2006), and anxiety (e.g., Derakshan et al., 2009), though it is still unknown whether disrupted inhibitory control is responsible for the abnormal emotional processing found in these disorders. Depressed patients, for example, are known to have deficient ability to inhibit processing of emotional stimuli (e.g., Goeleven et al., 2006) and thus, in this disorder the role of disrupted inhibition is clearer. (c) Considering the fact that inhibition is at least partially involved in most executive control tasks (including selective attention tasks), the connection between inhibition and emotion could potently contribute to the overall understating of the connection between executive control and emotion.

### The current study

The current study employed an emotional stop-signal task in order to examine the reciprocal links between emotion and inhibitory control. First, in no-stop-signal trials we predicted an emotional interference effect, similar to results obtained in simple discrimination tasks (Hartikainen et al., 2000; Buodo et al., 2002). Second, because we were using a design similar to the one used by Verbruggen and De Houwer (2007), we predicted a replication of their findings showing disrupted inhibitory control (i.e., longer SSRT) following negative compared to neutral pictures. Most importantly, we expected that the emotional interference would be eliminated when the previous trial was a stop trial. Namely, activation of inhibitory control processes during stop trials would reduce activation of negative stimuli on a following no-stop trial. This prediction was based on previous findings showing reduced emotional interference following executive activation using selective attention tasks (Cohen et al., 2011, 2012).

## Materials and methods

### Participants

Twenty-seven students of Ben-Gurion University of the Negev (Israel) participated for a small monetary payment. The study was approved by the ethical committee of the department of Psychology, Ben-Gurion University of the Negev, Israel. All participants signed an informed consent form previous to their participation in the experiment. All participants had normal or corrected-to-normal vision, were right-handed, had no history of attention deficit, or learning disabilities, and all were naive as to the purpose of the experiment. One participant was excluded from further analysis due to a high error rate on no-stop-signal trials [more than 3 standard deviations (*SD*) from the mean] and one was excluded due to report of severe depressive symptoms in a major depression inventory administered at the end of the behavioral task (MDI; Bech, 1997; Bech and Wermuth, 1998). In addition, because SSRT is an estimation of the time needed for a participant to stop on 50% of the trials, if a participant's success in inhibiting responses to stop trials was significantly different from 50%, the SSRT would not be valid and the participant would be excluded from further analysis [estimation method by Verbruggen and Logan (2009); see also Verbruggen et al. (2008)]. Three participants (females) were excluded due to the latter criterion. From the remaining 22 participants (10 females and 12 males) the youngest was 23 years old and the oldest was 29 years old (mean = 25.1 years, *SD* = 1.66).

### Equipment

Data collection and stimuli presentation were controlled by a DELL OptiPlex 760 vPro computer with an Intel core 2 duo processor E8400 3 GHz. Stimuli were presented on a DELL E198PF 19″ LCD monitor. A keyboard was placed on a table between the participant and the monitor. Participants were tested individually. They sat approximately 23.5 in. from the computer screen. Stickers with “@” and “#” signs were taped on two regular keyboard keys that served as response keys.

### Procedure

**Figure 1:**
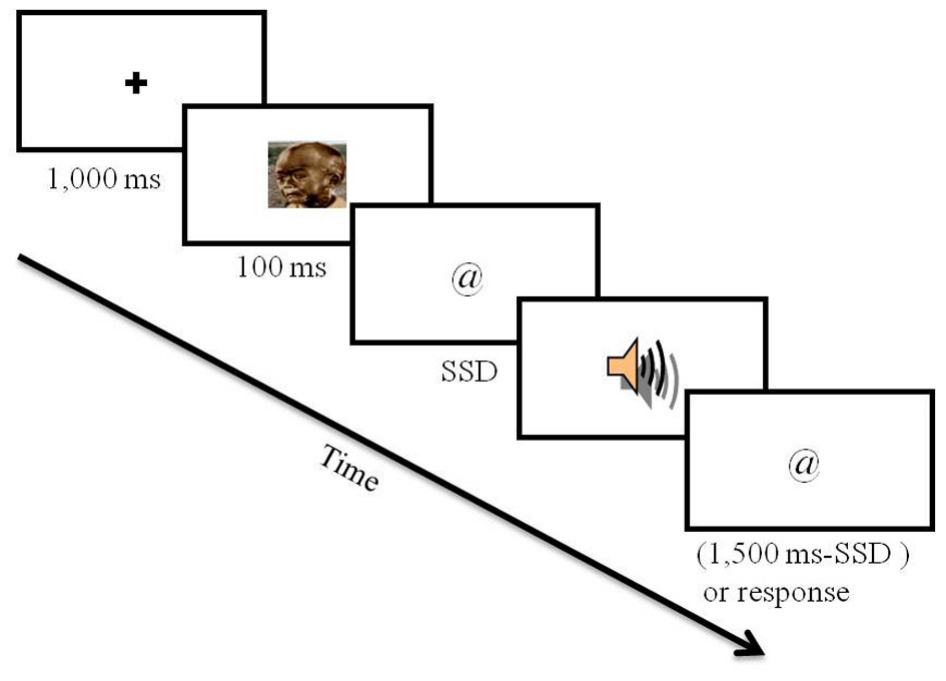
**Experimental procedure.** Example of a negative stop-signal trial.

The experiment included 12 practice trials, which were not further analyzed, and 480 experimental trials. Participants were told that the practice block would be identical to the experimental block, only that the experimental block would be longer and would not include feedback. Each trial started with a 1000 ms fixation (a black plus sign at the center of a gray screen). Fixation was followed by a picture for 100 ms. After the disappearance of the picture, a visual go stimulus appeared (i.e., @ or #). Response keys were “p” for the appearance of a “@” and “q” for a “#.” Participants were asked to respond with the index fingers of both hands. The instruction indicated to participants to press the correct key as fast and accurately as possible, and emphasized not to wait for a potential stop signal. The go stimulus stayed in view for 1500 ms or until a key press. RT was calculated from the appearance of the go stimulus to the response. On a random selection of 30% of the trials, an auditory stop signal was sounded (see Figure 1). The stop signal was presented after a variable SSD that was initially set at 250 ms and adjusted by a staircase tracking procedure: after each successful stopping the SSD was extended by 20 ms and after each unsuccessful stopping the SSD was shortened by 20 ms. In half of the trials a neutral picture was presented and in the other half a negative picture was presented. SSD was adjusted for each valence condition (i.e., negative and neutral) separately. Trial order was random with two restrictions: we had the same number of neutral and negative stop-signal trials (72 of each), and we had the same number of neutral and negative trials that followed stop-signal trials (36 of each valence condition in the current trial for each valance condition in the previous trial).

### Stimuli

Participants were presented with an emotional stop-signal task. We used 40 negative (mean valence = 2.41, mean arousal = 6.16) and 40 neutral (mean valence = 5.01, mean arousal = 2.84) pictures taken from the International Affective Picture System (IAPS; Lang et al., 2001). The pictures were identical to those used by Verbruggen and De Houwer (2007). Ten neutral pictures, different from those used in the actual experiment, were used in the practice phase. The go signals were black “@” or “#” signs presented at the center of a screen on a gray background and were 0.98 in. high and 2.36 in. wide. The stop signal was an auditory tone (750 Hz, 75 ms) delivered by headphones.

## Results

**Figure 2:**
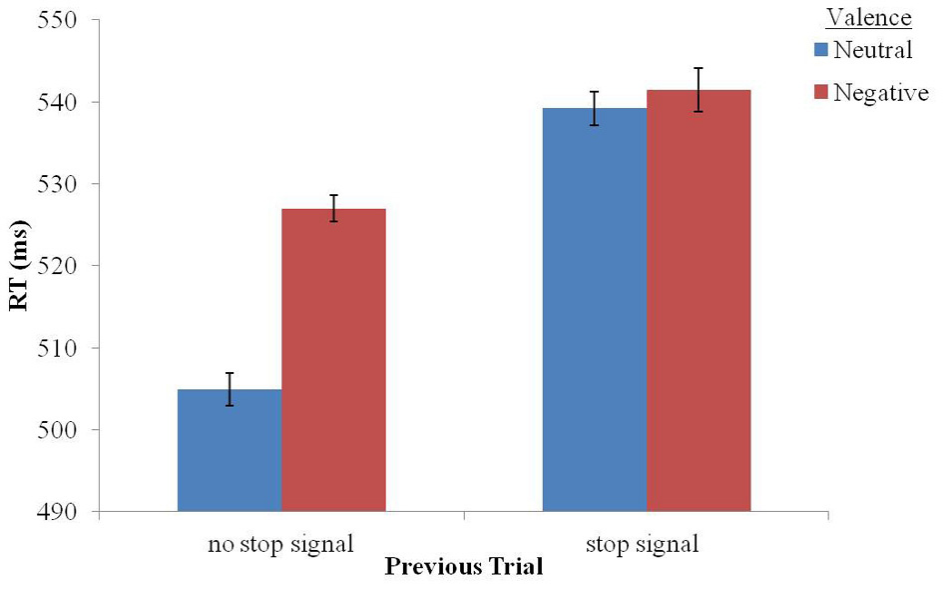
**Mean RT in the valence conditions following stop-signal trials or no stop-signal trials.** Error bars represent one standard error from the mean based on Cousineau's (2005) method for within-subjects designs.

In order to investigate our a-priori assumption that following stop-signal trials negative stimuli would not affect RT, a Two-Way analysis of variance (ANOVA) with repeated measures was applied to RT data of no-stop trials with valence (negative vs. neutral) and previous trial (no-stop vs. stop) as within-subject factors (see Table 1). A significant interaction between valence and previous trial was found, *F*_(1, 21)_ = 6.325, *p* < 0.02, *partial eta squared (PES)* = 0.231. As can be seen in Figure 2, following no-stop trials, RT for negative stimuli was significantly longer than RT for neutral stimuli, *F*_(1, 21)_ = 18.905, *p* < 0.001, *PES* = 0.474. In contrast, following stop trials, RT for negative stimuli did not differ significantly from RT for neutral trials, *F* < 1. Namely, the emotional interference effect was eliminated following stop-signal trials. This is similar to our previous findings showing attenuation of emotional interference following flanker incongruent trials (Cohen et al., 2011).

**Table 1:** **Reaction time (*RT in ms*), standard deviation (*SD*), and accuracy (*ACC*) of the different trials in the two valance conditions (Neutral and Negative)**.

	**Neutral**			**Negative**		
	***RT***	***SD***	***ACC***	***RT***	***SD***	***ACC***
No stop signal	517	69	0.97	533	67	0.97
Following no stop signal	505	75	0.96	527	71	0.97
Following stop signal	539	63	0.97	541	66	0.97
	***SSRT***	***SD***	***p(response/signal)***	***SSRT***	***SD***	***p(response/signal)***
Stop signal	187	59	0.49	210	62	0.46

In trials without a stop signal, mean RT of correct responses was calculated for each participant in each valence condition. A One-Way ANOVA with repeated measures was applied to RT data with valence (negative vs. neutral) as a within-subject factor (see Table 1). As expected, RT for negative stimuli was significantly longer than RT for neutral stimuli, *F*_(1, 21)_ = 22.191, *p* < 0.001, *PES* = 0.514. This finding replicates the known emotional interference effect, which was previously found by using simple discrimination tasks (Hartikainen et al., 2000; Buodo et al., 2002).

As mentioned before, SSD was adjusted for each valence condition separately. Based on the assumption that chances for successful inhibition were not significantly different than 0.50, SSRT was calculated as mean RT minus median SSD for each participant in each condition (see Verbruggen and Logan, 2009; see Table 1). As predicted, SSRT for negative trials was longer than SSRT for neutral trials. This was significant, *F*_(1, 21)_ = 4.301, *p* = 0.05, *PES* = 0.17. This result replicates Verbruggen and De Houwer's (2007) findings and strengthens the claim that emotional information disrupts inhibition-related executive functions.

## Discussion

The results of the current study are straightforward: first, emotional stimuli were found to impair responding and inhibitory control (i.e., elongated SSRT). Second, activation of inhibitory control was found to attenuate the (following) emotional effect.

In no stop-signal trials, responses to the discrimination task were slower when preceded by negative stimuli than when preceded by neutral stimuli; namely, we found an emotional interference effect (Hartikainen et al., 2000; Schimmack, 2005). This basically replicated previous findings that showed that negative stimuli disrupt performance in simple discrimination tasks that do not involve conflict (Hartikainen et al., 2000; Buodo et al., 2002). This finding corresponds with LeDoux's (1995) notion that emotional stimulus saliency is increased in order to enhance its processing.

Similar to Verbruggen and De Houwer (2007), we found that stopping latencies were prolonged following presentation of negative stimuli compared to stopping latencies following neutral stimuli. Namely, the ability to stop decreased when an irrelevant negative stimulus was presented. Similar to the results found in the no-stop trials, the findings showing elongated stopping latency following negative compared to neutral stimuli also strengthen the notion that emotional stimuli capture attention and receive prioritized processing compared to non-emotional stimuli. The idea that the presentation of a negative stimulus causes a momentary freeze (as would be expected from the fight, flight, freeze theory) can be interpreted in two ways by our findings. On the one hand, Verbruggen and De Houwer (2007) suggested that a momentary freeze should have helped stopping and thus SSRT should be shortened (improved) following a negative stimulus. On the other hand, a momentary cognitive freeze that occurs during the presentation of a negative stimulus would slow down the inhibitory control process. Our results, similar to those of Verbruggen and De Houwer, show slowdown both in the inhibitory control process and in RT to no-stop trials following a negative stimulus and thus indicate that cognitive freeze did occur. These results are in line with those of other studies that found reduced performance in executive tasks following emotional stimuli (e.g., Dennis et al., 2008; Padmala et al., 2011).

Importantly, in the current paper we examined the connection between briefly (100 ms) presented emotional stimuli (i.e., IAPS negative pictures) and inhibitory control. The findings reflect effects of a transient emotional arousal (as usually found when emotional and non-emotional pictures are presented randomly within the same block; Bradley et al., 1993) and not of a sustained emotional state or mood (as found when presenting a block of emotional pictures; Bradley et al., 1996). Briefly presented IAPS pictures are known to elicit emotional arousal as measured using physiological (Lang et al., 1993), electrophysiological (Schupp et al., 2004), and brain imaging (Glascher and Adolphs, 2003) methods (see Shackman et al., 2006 for a debate regarding assessment of emotional effects). In line with these findings, in the current study the IAPS pictures caused a momentary emotional arousal which affected performance of both go and stop processes.

The most important contribution of the current study is the finding regarding the effect of inhibitory control on emotion. The novelty of our study is that it examines whether inhibitory control can attenuate the effect of a following emotional stimulus. Our results show that while RT increased following negative stimuli, this effect disappeared in trials that where preceded by a stop-signal trial; namely, the emotional interference effect was not obtained following stop-signal trials. Accordingly, we suggest that the need to inhibit a pre-potent response activated inhibitory networks, which in turn down-regulated the emotional system and eliminated its influence on behavior. Given that the current research does not allow for direct measures of emotional processes, we cannot be sure whether inhibitory processes directly inhibits emotional processes or whether it inhibits the consequences of emotional processes. Further research is needed in order to investigate these interesting possibilities.

The current study's findings correspond with our previous findings that yielded a significant emotional interference effect after flanker-congruent trials but not after flanker-incongruent trials (Cohen et al., 2011). In that study it was argued that executive control activates top-down processes that can eliminate the influence of emotions on behavior. This regulatory connection was suggested as an interpretation for the finding that emotions did not affect executive control. To differentiate from that previous study, in the current study we found a “two-way” connection in which negative stimuli interrupted inhibitory control and operation of inhibitory control attenuated the influence of emotion on performance. As mentioned earlier, the flanker task and the stop-signal task activate different aspects of executive control (though there is some overlap between the mechanisms underlying them). Whereas the flanker task is mainly used to study selective attention or conflict control, the stop-signal task examines inhibitory control. While selective attention is characterize by the need to focus on the relevant stimulus or dimension and ignore irrelevant distracters, inhibitory control is characterize by the need to stop the current course of action. On the neurological level, selective attention tasks are associated mainly with activations of the anterior cingulate cortex and the dorsolateral prefrontal cortex (Cohen et al., 1990; Botvinick et al., 1999, 2001; Carter et al., 1999; Niendam et al., 2012), while stopping is mainly associated with activation of the rIFG; ventrolateral prefrontal cortex and the presupplementary motor area (pre-SMA) (Aron et al., 2003, 2007; Rubia et al., 2003; Chambers et al., 2007; Chevrier et al., 2007). It seems that the relationship between selective attention and emotion is not identical to the relationship between inhibitory control and emotion. Specifically, it seems that emotional stimuli impair inhibitory control but have inconsistent effects on selective attention. However, converging evidence from both of these executive components strengthens the notion that activation of executive control processes regulates the impact of emotion on behavior and on cognitive processes. As mentioned in the introduction, selective attention tasks activate inhibitory processes. It is possible that activation of the inhibitory control process underlies the top-down regulation effect found when using both selective attention and stop-signal tasks.

Some implications can be drawn from the current study results. Earlier, we mentioned that many psychopathologies and mood disorder are characterized by poor inhibitory control (e.g., autism—Geurts et al., 2004; schizophrenia—Enticott et al., 2008; obsessive-compulsive disorder—Chamberlain et al., 2006; and anxiety—Derakshan et al., 2009), and poor ability to suppress processing of emotional information (e.g., depression—Goeleven et al., 2006). Further research is needed in order to investigate the connection between the deficit in inhibitory control and the deficit in emotion regulation in these patients. Attention deficit\hyperactivity disorder (ADHD) is another widespread condition that the current study results may have implications for. People with ADHD are known to have deficient inhibitory control (stop-signal inhibition was proposed to be “an endophenotype of ADHD,” see Verbruggen and Logan, 2008, for review) and they also experience difficulties in emotion regulation (e.g., Walcott and Landau, 2006). The current study results imply that these two phenomena may be connected, though further research is needed in order to fully understand the connection between poor inhibitory control and the deficit in emotions regulation in individuals with ADHD.

To conclude, in the current study we demonstrated that emotional stimuli interfere with task performance, although, following trials that required inhibitory control this effect disappears—RT of negative trials was similar to RT of neutral trials. Additionally, we replicated previous findings showing that emotional stimuli interfere with inhibitory control. These findings suggest a two-way connection between inhibitory control and emotion in which emotion both disrupts and is modulated by inhibitory control. It seems that under some circumstances “high” cognitive systems can regulate or even suppress “low” systems such as the emotional system and thus prevent it from influencing performance. This mechanism has a potentially adaptive function—it enables goal-directed behavior in the presence of briefly presented irrelevant emotional information. Further research is still needed in order to uncover the specific circumstances in which this top-down regulation occurs and the implications of deficits in this regulation mechanism for emotion dysregulation disorders.

### Conflict of interest statement

The authors declare that the research was conducted in the absence of any commercial or financial relationships that could be construed as a potential conflict of interest.

## Footnotes

^1^In the context of this manuscript, we discuss the effects of briefly presented emotional stimuli (e.g., negative pictures). It is important to note that these stimuli cause temporary emotional arousal and do not create a long-lasting emotional state or mood.

## References

[B1] Aron A. R. Behrens T. E. Smith S. Frank M. J. Poldrack R. A. (2007). Triangulating a cognitive control network using diffusion weighted magnetic resonance imaging (MRI) and functional MRI. J. Neurosci. 27, 3743–3752 10.1523/JNEUROSCI.0519-07.2007 17409238 PMC6672420

[B2] Aron A. R. Fletcher P. C. Bullmore E. T. Sahakian B. J. Robbins T. W. (2003). Stop-signal inhibition disrupted by damage to right inferior frontal gyrus in humans. Nat. Neurosci. 6, 115–116 10.1038/nn1003 12536210

[B3] Banich M. T. (2009). Executive function: the search for an integrated account. Curr. Dir. Psychol. Sci. 18, 89–94

[B4] Bech P. (1997). Quality of life instruments in depression. Eur. Psychiatry 12, 194–198 10.1016/S0924-9338(97)89104-3 19698531

[B5] Bech P. Wermuth L. (1998). Applicability and validity of the Major Depression Inventory in patients with Parkinson's Disease. Nord. J. Psychiatry 52, 305–309

[B6] Bishop S. J. (2008). Neural mechanisms underlying selective attention to threat. Ann. N.Y. Acad. Sci. 1129, 141–152 10.1196/annals.1417.016 18591476

[B7] Blair K. S. Smith B. W. Mitchell D. G. Morton J. Vythilingam M. Pessoa L. (2007). Modulation of emotion by cognition and cognition by emotion. Neuroimage 35, 430–440 10.1016/j.neuroimage.2006.11.048 17239620 PMC1862681

[B8] Botvinick M. M. Braver T. S. Barch D. M. Carter C. S. Cohen J. D. (2001). Conflict monitoring and cognitive control. Psychol. Rev. 108, 624–652 11488380 10.1037/0033-295x.108.3.624

[B9] Botvinick M. M. Nystrom L. E. Fissell K. Carter C. S. Cohen J. D. (1999). Conflict monitoring versus selection-for-action in anterior cingulate cortex. Nature 402, 179–181 10.1038/46035 10647008

[B10] Bradley M. M. Cuthbert B. N. Lang P. J. (1996). Picture media and emotion: effects of a sustained affective context. Psychophysiology 33, 662–670 8961788 10.1111/j.1469-8986.1996.tb02362.x

[B11] Bradley M. M. Lang P. J. Cuthbert B. N. (1993). Emotion, novelty, and the startle reflex: habituation in humans. Behav. Neurosci. 107, 970–980 8136072 10.1037//0735-7044.107.6.970

[B12] Buodo S. Sarlo M. Palomba D. (2002). Attentional resources measured by reaction times highlight differences within pleasant and unpleasant, high arousing stimuli. Motiv. Emot. 26, 123–138

[B13] Carter C. S. Botvinick M. M. Cohen J. D. (1999). The contribution of the anterior cingulated cortex to executive processes in cognition. Rev. Neurosci. 10, 49–57 10356991 10.1515/revneuro.1999.10.1.49

[B14] Chamberlain S. R. Fineberg N. A. Blackwell A. D. Robbins T. W. Sahakian B. J. (2006). Motor inhibition and cognitive flexibility in obsessive-compulsive disorder and trichotillomania. Am. J. Psychiatry 163, 1282–1284 10.1176/appi.ajp.163.7.1282 16816237

[B15] Chambers C. D. Bellgrove M. A. Gould I. C. English T. Garavan H. McNaught E. (2007). Dissociable mechanisms of cognitive control in prefrontal and premotor cortex. J. Neurophysiol. 98, 3638–3647 10.1152/jn.00685.2007 17942624

[B16] Chevrier A. D. Noseworthy M. D. Schachar R. (2007). Dissociation of response inhibition and performance monitoring in the stop signal task using event-related fMRI. Hum. Brain Mapp. 28, 1347–1358 10.1002/hbm.20355 17274022 PMC6871417

[B17] Cohen J. D. Dunbar K. McClelland J. L. (1990). On the control of automatic processes: a parallel distributed processing account of the Stroop effect. Psychol. Rev. 97, 332–361 2200075 10.1037/0033-295x.97.3.332

[B18] Cohen N. Henik A. (2012). Do irrelevant emotional stimuli impair or improve executive control? Front. Integr. Neurosci. 6:33 10.3389/fnint.2012.00033 22719722 PMC3376948

[B19] Cohen N. Henik A. Mor N. (2011). Can emotion modulate attention? Evidence for reciprocal links in the Attentional Network Test. Exp. Psychol. 58, 171–179 10.1027/1618-3169/a000083 20705545

[B20] Cohen N. Henik A. Moyal N. (2012). Executive control attenuates emotional effects—For high reappraisers only? Emotion 12, 970–979 10.1037/a0026890 22251044

[B21] Cousineau D. (2005). Confidence intervals in within-subjects designs: a simpler solution to Loftus and Masson's method. Tutorial Quant. Methods Psychol. 1, 42–45 10.3758/s13423-012-0230-1 22441956 PMC3348489

[B22] Dennis T. A. Chen C. C. McCandliss B. D. (2008). Threat related attentional biases: an analysis of three attention systems. Depress. Anxiety 25, 1–10 10.1002/da.20308 17565734 PMC2662699

[B23] Derakshan N. Ansari T. L. Hansard M. Shoker L. Eysenck M. W. (2009). Anxiety, inhibition, efficiency, and effectiveness: an investigation using the antisaccade task. Exp. Psychol. 56, 48–55 10.1027/1618-3169.56.1.48 19261578

[B24] Enticott P. G. Ogloffa J. R. P. Bradshaw J. L. (2008). Response inhibition and impulsivity in schizophrenia. Psychiatry Res. 157, 251–254 10.1016/j.psychres.2007.04.007 17916385

[B25] Eriksen B. A. Eriksen C. W. (1974). Effects of noise letters upon the identification of a target letter in a nonsearch task. Percept. Psychophys. 16, 143–149

[B26] Etkin A. Egner T. Peraza D. M. Kandel E. R. Hirsch J. (2006). Resolving emotional conflict: A role for the rostral anterior cingulate cortex in modulating activity in the amygdala. Neuron 51, 871–882 10.1016/j.neuron.2006.07.029 16982430

[B27] Etkin A. Prater K. E. Hoeft F. Menon V. Schatzberg A. F. (2010). Failure of anterior cingulate activation and connectivity with the amygdala during implicit regulation of emotional processing in generalized anxiety disorder. Am. J. Psychiatry 167, 545–554 10.1176/appi.ajp.2009.09070931 20123913 PMC4367202

[B28] Geurts H. M. Verté S. Oosterlaan J. Roeyers H. Sergeant J. A. (2004). How specific are executive functioning deficits in attention deficit hyperactivity disorder and autism? J. Child Psychol. Psychiatry 45, 836–854 10.1111/j.1469-7610.2004.00276.x 15056314

[B29] Glascher J. Adolphs R. (2003). Processing of the arousal of subliminal and supraliminal emotional stimuli by the human amygdala. J. Neurosci. 23, 10274–10282 14614086 10.1523/JNEUROSCI.23-32-10274.2003 PMC6741000

[B30] Goeleven E. De Raedt R. Baert S. Koster E. H. W. (2006). Deficient inhibition of emotional information in depression. J. Affect. Disord. 93, 1–3 10.1016/j.jad.2006.03.007 16647141

[B31] Hariri A. Bookheimer S. Mazziotta J. (2000). Modulating emotional responses: effects of a neocortical network on the limbic system. Neuroreport 11, 43–48 10683827 10.1097/00001756-200001170-00009

[B32] Harnishfeger K. K. (1995). The development of cognitive inhibition: theories, definitions, and research evidence, in Interference and Inhibition in Cognition, eds Dempster F. N. Brainerd C. J. (San Diego, CA: Academic Press), 175–204

[B33] Hart S. J. Green S. R. Casp M. Belger A. (2010). Emotional priming effects during Stroop task performance. Neuroimage 49, 2662–2670 10.1016/j.neuroimage.2009.10.076 19883772 PMC2818423

[B34] Hartikainen K. M. Ogawa K. H. Knight R. T. (2000). Transient interference of right hemispheric function due to automatic emotional processing. Neuropsychologia 38, 1576–1580 10.1016/S0028-3932(00)00072-5 11074080

[B35] Kalanthroff E. Goldfarb L. Henik A. (2012). Evidence for interaction between the Stop-Signal and the Stroop task conflict. J. Exp. Psychol. Hum. Percept. Perform. [Epub ahead of print]. 10.1037/a0027429 22390293

[B36] Kanske P. (2012). On the influence of emotion on conflict processing. Front. Integr. Neurosci. 6:42 10.3389/fnint.2012.00042 22876220 PMC3410615

[B37] Kanske P. Kotz S. A. (2010). Modulation of early conflict processing: N200 responses to emotional words in a flanker task. Neuropsychologia 48, 3661–3664 10.1016/j.neuropsychologia.2010.07.021 20654636

[B38] Kanske P. Kotz S. A. (2011a). Emotion speeds up conflict resolution: a new role for the ventral anterior cingulate cortex? Cereb. Cortex 21, 911–919 10.1093/cercor/bhq157 20732901

[B39] Kanske P. Kotz S. A. (2011b). Positive emotion speeds up conflict processing: ERP responses in an auditory Simon task. Biol. Psychol. 87, 122–127 10.1016/j.biopsycho.2011.02.018 21382438

[B40] Lang P. J. Bradley M. M. Cuthbert B. N. (2001). International affective picture system (IAPS): instruction manual and affective ratings, in Technical Report A-5, (Gainesville, FL: The Center for Research in Psychophysiology, University of Florida).

[B41] Lang P. J. Greenwald M. K. Bradley M. M. Hamm A. O. (1993). Looking at pictures: affective, visceral, and behavioral reactions. Psychophysiology 30, 261–273 8497555 10.1111/j.1469-8986.1993.tb03352.x

[B42] LeDoux J. (1995). Emotion—clues from the brain. Ann. Rev. Psychol. 46, 209–235 10.1146/annurev.ps.46.020195.001233 7872730

[B43] Liberzon I. Taylor S. Fig L. Decker L. Koeppe R. Minoshima S. (2000). Limbic activation and psychophysiologic responses to aversive visual stimuli: interaction with cognitive task. Neuropsychopharmacology 23, 508–516 10.1016/S0893-133X(00)00157-3 11027916

[B44] Logan G. D. (1994). On the ability to inhibit thought and action: a user's guide to the stop signal paradigm, in Inhibitory Processes in Attention, Memory and Language, eds Dagenbach D. Carr T. H. (San Diego, CA: Academic Press), 189–239

[B45] Logan G. D. Cowan W. B. (1984). On the ability to inhibit thought and action: a theory of an act of control. Psychol. Rev. 91, 295–327 10.1016/j.neubiorev.2008.08.014 24490789

[B46] Logan G. D. Cowan W. B. Davis W. B. (1984). On the ability to inhibit simple and choice reaction time responses: a model and a method. J. Exp. Psychol. Hum. Percept. Perform. 10, 276–291 6232345 10.1037//0096-1523.10.2.276

[B47] MacLeod C. M. (1991). Half a century of research on the Stroop effect: an integrative review. Psychol. Bull. 109, 163–203 2034749 10.1037/0033-2909.109.2.163

[B48] Miller E. K. Cohen J. D. (2001). An integrative theory of prefrontal cortex function. Ann. Rev. Neurosci. 24, 167–202 10.1146/annurev.neuro.24.1.167 11283309

[B49] Mitchell D. Luo Q. Mondillo K. Vythilingam M. Finger E. Blair R. (2008). The interference of operant task performance by emotional distracters: an antagonistic relationship between the amygdala and frontoparietal cortices. Neuroimage 40, 859–868 10.1016/j.neuroimage.2007.08.002 18234519 PMC2693278

[B50] Miyake A. Friedman N. P. Emerson M. J. Witzki A. H. Howerter A. Wager T. D. (2000). The unity and diversity of executive functions and their contributions to complex “frontal lobe” tasks: a latent variable analysis. Cogn. Psychol. 41, 49–100 10.1006/cogp.1999.0734 10945922

[B51] Niendam T. A. Laird A. R. Ray K. L. Dean Y. M. Glahn D. C. Carter C. S. (2012). Meta-analytic evidence for a superordinate cognitive control network subserving diverse executive functions. Cogn. Affect. Behav. Neurosci. 12, 241–268 10.3758/s13415-011-0083-5 22282036 PMC3660731

[B52] Nigg J. T. (2000). On inhibition/disinhibition in developmental psychopathology: views from cognitive and personal psychology and a working inhibition taxonomy. Psychol. Bull. 126, 1–27 10748641 10.1037/0033-2909.126.2.220

[B53] Ochsner K. N. Gross J. J. (2005). The cognitive control of emotion. Trends Cogn. Sci. 9, 242–249 10.1016/j.tics.2005.03.010 15866151

[B54] Okon-Singer H. Lichtenstein-Vidne L. Cohen N. (2012). Dynamic modulation of emotional processing. Biol. Psychol. [Epub ahead of print]. 10.1016/j.biopsycho.2012.05.010 22676964

[B55] Padmala S. Bauer A. Pessoa L. (2011). Negative emotion impairs conflict-driven executive control. Front. Psychol. 2:192 10.3389/fpsyg.2011.00192 21886635 PMC3154405

[B56] Pessoa L. (2005). To what extent are emotional visual stimuli processed without attention and awareness? Curr. Opin. Neurobiol. 15, 188–196 10.1016/j.conb.2005.03.002 15831401

[B57] Pessoa L. (2009). How do emotion and motivation direct executive control? Trends Cogn. Sci. 13, 160–166 10.1016/j.tics.2009.01.006 19285913 PMC2773442

[B58] Pessoa L. Padmala S. Kenzer A. Bauer A. (2012). Interactions between cognition and emotion during response inhibition. Emotion 12, 192–197 10.1037/a0024109 21787074 PMC3208031

[B59] Posner M. I. Petersen S. E. (1990). The attention system of the human brain. Annu. Rev. Neurosci. 13, 25–42 10.1146/annurev.ne.13.030190.000325 2183676

[B60] Rafal R. Henik A. (1994). The neurology of inhibition: integrating controlled and automatic processes, in Inhibitory Processes in Attention, Memory, and Language, eds Dagenbach D. Carr T. H. (San Diego, CA: Academic Press), 1–51

[B61] Rubia K. Smith A. B. Brammer M. J. Taylor E. (2003). Right inferior prefrontal cortex mediates response inhibition while mesial prefrontal cortex is responsible for error detection. Neuroimage 20, 351–358 10.1016/S1053-8119(03)00275-1 14527595

[B62] Sagaspe P. Schwartz S. Vuilleumier P. (2011). Fear and stop: a role for the amygdala in motor inhibition by emotional signals. Neuroimage 55, 1825–1835 10.1016/j.neuroimage.2011.01.027 21272655

[B63] Schimmack U. (2005). Attentional interference effects of emotional pictures: threat, negativity or arousal? Emotion 5, 55–66 10.1037/1528-3542.5.1.55 15755219

[B64] Schupp H. T. Junghofer M. Weike A. I. Hamm A. O. (2004). The selective processing of briefly presented affective pictures: an ERP analysis. Psychophysiology 41, 441–449 10.1111/j.1469-8986.2004.00174.x 15102130

[B65] Shackman A. J. Sarinopolulos I. Maxwell J. S. Pizzagalli D. Lavric A. Davidson R. J. (2006). Anxiety selectively disrupts visuospatial working memory. Emotion 6, 40–61 10.1037/1528-3542.6.1.40 16637749

[B66] Shallice T. Norman D. (1986). Attention to action: willed and automatic control of behavior, in Consciousness and Self-Regulation: Advances in Research and Theory, Vol. 4, eds Davidson R. Schwartz G. Shapiro D. (New York, NY: Plenum Press), 1–18

[B67] Stroop J. R. (1935). Studies of interference in serial verbal reactions. J. Exp. Psychol. 18, 643–662

[B68] van Veen V. Carter C. S. (2006). Conflict and cognitive control in the brain. Curr. Dir. Psychol. Sci. 15, 237–240 10.1017/S1355617709990701 19765356

[B69] Verbruggen F. De Houwer J. (2007). Do emotional stimuli interfere with response inhibition? Evidence from the stop signal paradigm. Cogn. Emot. 21, 391–403

[B70] Verbruggen F. Liefooghe B. Vandierendonck A. (2004). The interaction between stop signal inhibition and distractor interference in the flanker and the Stroop task. Acta Psychol. 116, 21–37 10.1016/j.actpsy.2003.12.011 15111228

[B71] Verbruggen F. Logan G. (2008). Response inhibition in the stop-signal paradigm. Trends Cogn. Sci. 12, 418–424 10.1016/j.tics.2008.07.005 18799345 PMC2709177

[B72] Verbruggen F. Logan G. (2009). Models of response inhibition in the stop-signal and stop-change paradigms. Neurosci. Biobehav. Rev. 33, 647–661 10.1016/j.neubiorev.2008.08.014 18822313 PMC2696813

[B73] Verbruggen F. Logan G. D. Stevens M. A. (2008). STOP-IT: windows executable software for the stop-signal paradigm. Behav. Res. Methods 40, 479–483 18522058 10.3758/brm.40.2.479

[B74] Vuilleumier P. (2005). How brains beware: neural mechanisms of emotional attention. Trends Cogn. Sci. 9, 585–594 10.1016/j.tics.2005.10.011 16289871

[B75] Walcott C. M. Landau S. (2006). The relation between disinhibition and emotion regulation in boys with attention deficit hyperactivity disorder. J. Clin. Child. Adolesc. Psychol. 33, 772–782 10.1207/s15374424jccp3304_12 15498744

